# Eosinophilic Annular Erythema Associated With IgM Monoclonal Gammopathy of Undetermined Significance

**DOI:** 10.7759/cureus.104220

**Published:** 2026-02-25

**Authors:** Shin Iinuma, Takahiro Kobayashi, Takahiro Nagashima, Yasuyuki Fujita

**Affiliations:** 1 Dermatology, Japanese Red Cross Kitami Hospital, Kitami, JPN; 2 Dermatology, Asahikawa Medical University, Asahikawa, JPN; 3 Internal Medicine, Japanese Red Cross Kitami Hospital, Kitami, JPN

**Keywords:** eosinophilic annular erythema, eosinophilic dermatosis of hematologic malignancy, lymphoplasmacytic lymphoma, monoclonal gammopathy, waldenström macroglobulinemia

## Abstract

Eosinophilic annular erythema (EAE) is a rare inflammatory skin disease characterized by recurrent annular or figurate erythematous plaques and eosinophil-rich dermal infiltrates. Here, we present the case of a 73-year-old Japanese woman who had a 10-month history of pruritic annular and figurate plaques with central postinflammatory hyperpigmentation of the trunk and proximal extremities. Histopathological examination revealed dense perivascular and interstitial lymphocytic infiltrates with numerous eosinophils in the dermis, without vasculitis or flame figures, consistent with EAE. Laboratory evaluation revealed markedly elevated serum IgM, and immunofixation demonstrated an IgM-λ monoclonal protein with urinary λ-type Bence Jones protein. She was asymptomatic and had no evidence of end-organ damage, including hypercalcemia, renal insufficiency, anemia, or bone lesions. Additionally, the hematologic findings were consistent with a diagnosis of IgM monoclonal gammopathy of undetermined significance. The eruption regressed with high-potency topical corticosteroids, and the patient was managed with dermatological therapy and hematological surveillance. Reports of EAE associated with IgM monoclonal gammopathy are scarce. This case highlights that EAE may coexist with IgM monoclonal gammopathy and supports hematological evaluation and follow-up when IgM monoclonal proteins are detected.

## Introduction

Eosinophilic annular erythema (EAE) is a rare inflammatory dermatosis clinically characterized by recurrent annular or figurate erythematous plaques. Central pigmentation corresponding to basal melanosis is a characteristic feature, and histopathological examination typically reveals an eosinophil-rich perivascular and interstitial dermal infiltrate [1]. Since EAE spectrum overlaps with that of eosinophilic cellulitis (Wells syndrome) and other eosinophilic dermatoses, establishing a diagnosis can be challenging, and careful correlation between clinical and histopathological findings is required. Although EAE is frequently idiopathic, accumulating reports suggest associations with systemic conditions, including autoimmune diseases, infections, and hematological disorders [2].

IgM monoclonal gammopathy spans the spectrum from IgM monoclonal gammopathy of undetermined significance (IgM MGUS) to lymphoplasmacytic lymphoma/Waldenström macroglobulinemia (LPL/WM) [3]. IgM MGUS is a premalignant, typically indolent clonal condition. It is defined by the presence of an IgM monoclonal protein without end-organ damage or other treatment-requiring features and may precede LPL/WM. Accordingly, identification of IgM monoclonal proteins should prompt hematological evaluation and longitudinal follow-up to assess progression along this spectrum. Moreover, IgM MGUS has been associated with paraneoplastic or immune-mediated cutaneous manifestations, and dermatological findings may occasionally provide an early clue to an underlying clonal B-cell disorder. In this report, we describe a case of EAE in a patient with concomitant IgM MGUS, highlighting the importance of recognizing EAE-like eruptions as a potential indicator of monoclonal gammopathy.

## Case presentation

**Figure 1:**
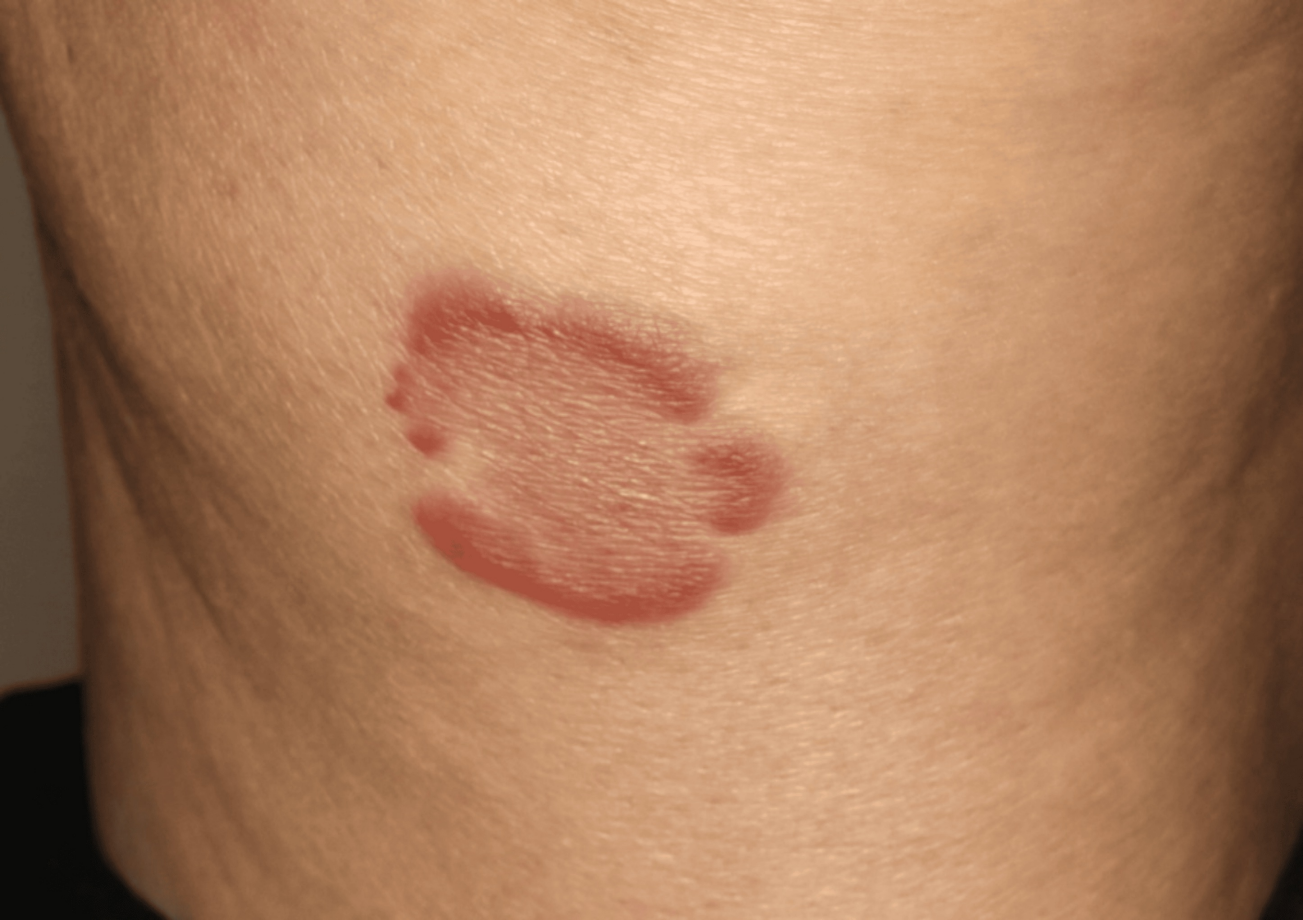
Clinical presentation showing a well-demarcated erythematous annular plaque with central postinflammatory hyperpigmentation on the right medial thigh

**Figure 2:**
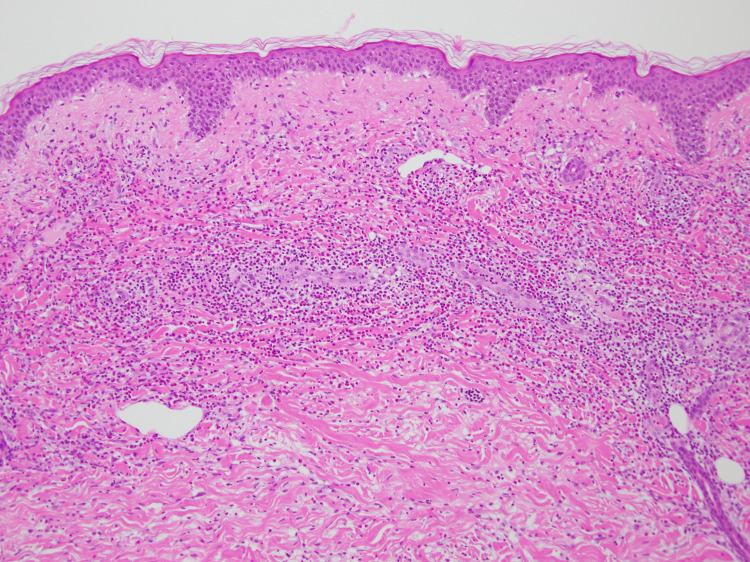
Histopathology (low magnification) showing a dense superficial-to-deep perivascular and interstitial inflammatory infiltrate within the dermis (hematoxylin and eosin staining, original magnification ×100)

**Figure 3:**
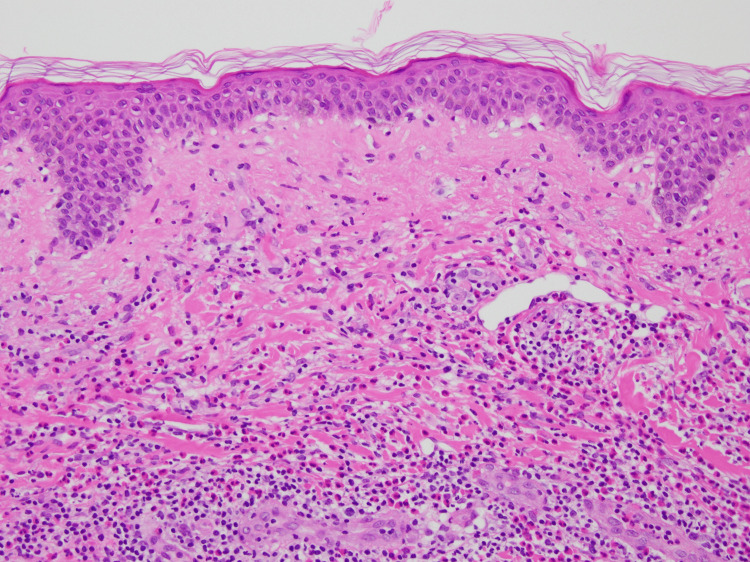
Histopathology (high magnification) showing numerous eosinophils admixed with lymphocytes in a perivascular and interstitial pattern in the dermis, with mild basal melanosis and vacuolar change. Neither vasculitis nor flame figures are identified (hematoxylin and eosin, original magnification ×200)

A 73-year-old Japanese woman presented to our dermatology department with recurrent pruritic eruptions. She had first noticed erythematous lesions that gradually expanded centrifugally on the trunk and proximal extremities approximately 10 months prior to presentation. The patient’s medical history was unremarkable. Physical examination revealed variably sized erythematous annular and figurate plaques, with diameters ranging from approximately 3 to 10 cm, as well as central postinflammatory hyperpigmentation on the trunk and proximal extremities (Figure 1). A 4-mm punch biopsy obtained from a representative lesion showed mild basal melanosis with vacuolar changes and dense perivascular and interstitial lymphocytic infiltrates with numerous eosinophils in the dermis, with no extension into the subcutaneous tissue (Figures 2, 3). This dermis-limited eosinophil-rich infiltrate supported EAE and helped distinguish it from Wells syndrome and eosinophilic dermatosis of hematologic malignancy (EDHM), in which eosinophil-rich inflammation more often extends into the subcutis. Neither vasculitis nor flame figures, characteristic of Wells syndrome, were observed.

Initial laboratory testing revealed a white blood cell count of 5.97 × 10⁹/L, hemoglobin level of 11.8 g/dL, and platelet count of 288 × 10⁹/L, with the following differential counts: neutrophils 63%, lymphocytes 27.1%, monocytes 8.2%, eosinophils 1.2%, and basophils 0.5% (absolute eosinophil count, 0.07 × 10⁹/L). Serum biochemistry results were unremarkable, and C-reactive protein level was mildly increased (4.8 mg/L; reference range <1.4 mg/L). Autoimmune serology findings were also unremarkable, except for an antinuclear antibody titer of 1:80 (<1:40) and normal complement levels. Myeloperoxidase antineutrophil cytoplasmic antibody (MPO-ANCA), anti-Sjögren's-syndrome-related antigen A antibody, and anti-BP180 antibody test results were all negative. Serum immunoglobulin testing showed markedly elevated IgM levels (2,161.4 mg/dL; 50-269 mg/dL), with low IgG (757 mg/dL; 861-1,747 mg/dL) and low IgE (<25 IU/mL; <358 IU/mL). Soluble interleukin-2 receptor levels were elevated (1,805 U/mL; 122-496 U/mL). The patient was referred to the hematology department for further evaluation.

Serum immunofixation demonstrated an IgM-λ monoclonal protein, and urinary testing identified λ-type Bence Jones protein. The IgM M-protein levels were <3.0 g/dL. Cold agglutinin and cryoglobulin tests and screening tests for chronic infections yielded negative results. Contrast-enhanced computed tomography revealed no significant lymphadenopathy, hepatosplenomegaly, lytic bone lesions, or soft-tissue masses. Bone marrow aspiration revealed plasma cells comprising 3.2% of nucleated cells, with <10% small atypical lymphocytes. Flow cytometry demonstrated a light-chain-restricted B-cell population (CD10−, CD19+, CD20+), with CD5 negative and CD23 indeterminate. The patient was asymptomatic and showed no evidence of end-organ damage (hypercalcemia, renal insufficiency, anemia, or bone lesions (CRAB)). Overall, these findings were consistent with IgM MGUS, and LPL/WM was considered unlikely given the limited marrow involvement, although a very early stage could not be entirely excluded.

The patient was diagnosed with EAE in the setting of concomitant IgM-MGUS. As there were no hematological complications requiring immediate treatment, she was managed with close observation. Dermatologically, the eruptions tended to regress with high-potency topical corticosteroid therapy (clobetasol propionate ointment). Each flare began to improve within two weeks and resolved within four weeks, with intermittent topical corticosteroid use as needed thereafter during three years of combined dermatologic and hematologic follow-up.

## Discussion

Our case highlights a patient with recurrent annular and figurate erythematous plaques and an eosinophil-rich perivascular and interstitial dermal infiltrate without vasculitis or flame figures, consistent with EAE, in the setting of concomitant IgM MGUS. EAE has generally been regarded as an idiopathic eosinophil-predominant dermatosis; however, accumulating evidence indicates that it may coexist with hematological disorders. In a French multicenter retrospective series of 18 patients, hematologic malignancies were present in four (22.2%), including polycythemia vera (n = 2) and B-cell lymphoma (n = 2), indicating that a subset of adult EAE occurs in association with clonal hematologic disease [2]. While these observations do not prove causality, they raise the possibility that EAE-like eruptions may reflect immune dysregulation related to an underlying clonal B-cell process. Therefore, evaluation for an associated hematologic disorder, including monoclonal gammopathy, may be clinically warranted when EAE is diagnosed, particularly in recurrent cases. Notably, peripheral eosinophilia is not required for tissue eosinophilia, and localized chemokine-driven recruitment may explain a normal blood eosinophil count despite prominent dermal eosinophilic infiltration.

The principal differential diagnoses include eosinophilic cellulitis, eosinophilic granulomatosis with polyangiitis (EGPA), Schnitzler’s syndrome, and early (nonbullous) bullous pemphigoid. Eosinophilic cellulitis may present with erythematous plaques and tissue eosinophilia, and histopathology typically shows eosinophil-rich inflammation extending into the subcutis with flame figures. Notably, our case showed no flame figures, and the eosinophil-rich infiltrate was confined to the dermis, supporting EAE rather than eosinophilic cellulitis [1]. EGPA was also considered; however, there were no clinical features suggestive of systemic vasculitis, neuropathy, or peripheral eosinophilia, and MPO-ANCA was negative [4]. Schnitzler’s syndrome was also considered, given the concomitant IgM MGUS, but the eruption consisted of persistent annular or polycyclic plaques rather than chronic urticaria. Moreover, Schnitzler-associated urticaria is often neutrophil-predominant, whereas our case demonstrated an eosinophil-rich infiltrate and lacked systemic inflammatory manifestations, such as recurrent fever, bone pain, or markedly increased inflammatory markers [5]. Early (nonbullous) bullous pemphigoid was considered because its prodromal phase can mimic other inflammatory dermatoses. However, the anti-BP180 antibody test was negative, and histopathology showed neither eosinophilic spongiosis nor subepidermal blistering, arguing against pemphigoid at presentation [6].

Given the association between eosinophilic skin diseases and B-cell lymphoproliferative disorders, EDHM is an important diagnostic consideration. Histopathologically, EDHM typically shows eosinophil-rich dermal infiltrates, often with superficial and deep perivascular and interstitial distributions, which may be accompanied by epidermal changes such as spongiosis or vesiculation [7,8]. Clinically, EDHM presents as a pruritic relapsing eruption with a heterogeneous morphology, including papules, urticarial plaques, vesicles, or insect bite-like lesions. In contrast, our patient showed a more uniform annular and figurate morphology and an eosinophil-rich infiltrate confined to the dermis without vesiculation. Nevertheless, clinicopathological overlap among eosinophilic dermatoses is possible, and EDHM should remain a differential diagnosis when eosinophil-predominant eruptions arise in clonal B cell disorders.

From the hematologic perspective, our patient had IgM-λ monoclonal proteins with urinary λ-type Bence Jones protein and a minor light-chain-restricted B-cell population in the bone marrow, without significant lymphadenopathy, organomegaly, or treatment-requiring complications. She was asymptomatic and did not meet the CRAB criteria [9]. The serum IgM M-protein levels were <3 g/dL, and bone marrow lymphoplasmacytic involvement was <10%, supporting IgM MGUS. Nevertheless, a very early stage of LPL/WM could not be entirely excluded. Since IgM monoclonal gammopathy exists on the spectrum of LPL/WM, a standardized hematologic workup is essential for accurate classification and risk stratification [10]. IgM MGUS carries a persistent risk of progression to LPL/WM of approximately 1% per year [11]; therefore, detection of IgM monoclonal proteins in patients with eosinophilic dermatoses should prompt hematological evaluation and longitudinal follow-up. If a cutaneous eruption flares or becomes refractory, repeat hematological reassessment may also be warranted.

The management of EAE has not been standardized; however, the most used and effective therapies include topical or systemic corticosteroids, hydroxychloroquine, and dapsone [2]. In refractory cases, combination regimens and emerging targeted options, such as biologics or Janus kinase inhibitors, have also been reported [12]. Although some patients respond well to topical corticosteroids alone, relapses are common. Therefore, escalation to systemic therapy should be considered according to disease severity and recurrence.

## Conclusions

We report a case of EAE associated with IgM-MGUS. Although a causal relationship cannot be established, this case suggests that EAE may coexist with IgM monoclonal gammopathy and may serve as a clinical clue for an underlying clonal hematologic disorder. Therefore, when an IgM monoclonal protein is identified in patients with eosinophilic annular eruptions, hematologic evaluation and longitudinal follow-up should be considered while maintaining a broad differential diagnosis.
